# Measles infection causing Bacillus Calmette-Guérin reactivation: a case report

**DOI:** 10.1186/s12887-019-1635-z

**Published:** 2019-07-24

**Authors:** Sobana Muthuvelu, Kev Shiau-Chong Lim, Ling-Yin Huang, Shi-Tying Chin, Anand Mohan

**Affiliations:** 1 Department of Paediatrics, Bintulu Hospital, Bintulu, Sarawak Malaysia; 2 Department of Paediatrics, Sungai Buloh Hospital, Sungai Buloh, Selangor Malaysia; 3 Department of Paediatrics, Bintulu Medical Centre, Bintulu, Sarawak Malaysia

**Keywords:** Measles, Bacillus Calmette-Guérin, Reactivation, Kawasaki disease

## Abstract

**Background:**

Reactivation of the Bacillus Calmette-Guérin (BCG), manifesting as erythema, induration, ulceration or crust formation at a previous BCG inoculation site, is a common and highly specific feature of Kawasaki disease (KD). We report the unusual finding of BCG reactivation in an infant with laboratory-confirmed measles.

**Case presentation:**

A previously healthy 7-month old infant presented initially with fever, cough and coryza, and subsequently developed Koplik’s spots followed by a typical morbilliform skin rash. There was significant contact history with a household relative who had recently been diagnosed with measles. On examination, a 2.5 cm area of erythema and induration was seen at the previous BCG inoculation site, in addition to the widespread maculopapular rash. No other clinical features of KD were present. Measles virus was isolated from the throat swab and measles antibodies (IgM) were present in the serum. The patient recovered completely with oral vitamin A and supportive therapy, and had normal echocardiography examination on follow up.

**Conclusions:**

This case report highlights the rare finding of BCG reactivation in a child with confirmed measles infection, and suggests that this clinical manifestation may occasionally occur in children with infections or conditions other than KD.

## Background

Bacillus Calmette-Guérin (BCG) vaccine is used to prevent tuberculous meningitis, miliary tuberculosis and possibly other forms of tuberculosis, including pulmonary disease, in children [1, 2]. It is included as part of the expanded programme on immunization in over 150 countries worldwide, including Malaysia [3]. In Malaysia, as in most countries where it is used, BCG vaccination is recommended universally for all infants at birth or shortly after birth. Following intradermal inoculation of the attenuated *Mycobacterium bovis* BCG strain at the deltoid region of the left arm, a small papule usually appears after 2 weeks, gradually enlarges and typically ulcerates within 2–4 months, and slowly heals over the next few months to form the BCG scar [4].

Reactivation of the BCG, manifesting as erythema, induration, ulceration or crust formation at the BCG site months or years after inoculation, has been described as an important feature of Kawasaki disease (KD) [5, 6]. Although not included in the diagnostic criteria of the disease, this clinical manifestation is reported in up to 50% of children with KD [7]. In addition, the clinical finding of BCG reactivation is regarded to be highly specific for KD, as it is very rarely described in other febrile illnesses or infections. For example, in a study of febrile Japanese children aged < 2 years admitted over an 8-month period with diagnoses other than KD, no BCG reactivations were observed [8]. In contrast, data from a Japanese nationwide epidemiologic survey showed that > 70% of children in this age group who were diagnosed with KD developed BCG reactivation [8]. Measles infection has not been reported to cause BCG reactivation.

Here, we present a case report of a 7-month old infant with laboratory-confirmed measles who presented with erythema and induration at the BCG inoculation site.

## Case presentation

A 7-month old boy was admitted to the paediatric ward of Bintulu Hospital in Sarawak with a 4-day history of fever and 1 day of rash. His past medical history had been unremarkable and he had been thriving well. He had received BCG vaccination (BCG-Japan, Tokyo 172 strain) at birth, at the same hospital. According to his mother, a papule had developed at the inoculation site which had then healed completely to form a scar in the months prior to the admission. He had not received measles vaccination.

His illness began abruptly with high fever, cough and coryza, for which he received symptomatic treatment as an outpatient at a private medical centre during the first 2 days after disease-onset. On the third day, his condition worsened as he fed poorly and was noted to have reduced activity, with persistence of the fever. A detailed history was then taken and revealed that the child’s uncle, who lives with the family and had never received measles vaccination, had been admitted to the same medical centre 2 weeks earlier with a laboratory-confirmed diagnosis of measles. Physical examination of the child revealed Koplik’s spots and he was then admitted to the medical centre. Later that day, he developed a typical morbilliform rash which began behind the ears and neck, and spread to his face and trunk. The following day, he was referred to our hospital with a clinical diagnosis of measles.

**Fig. 1:**
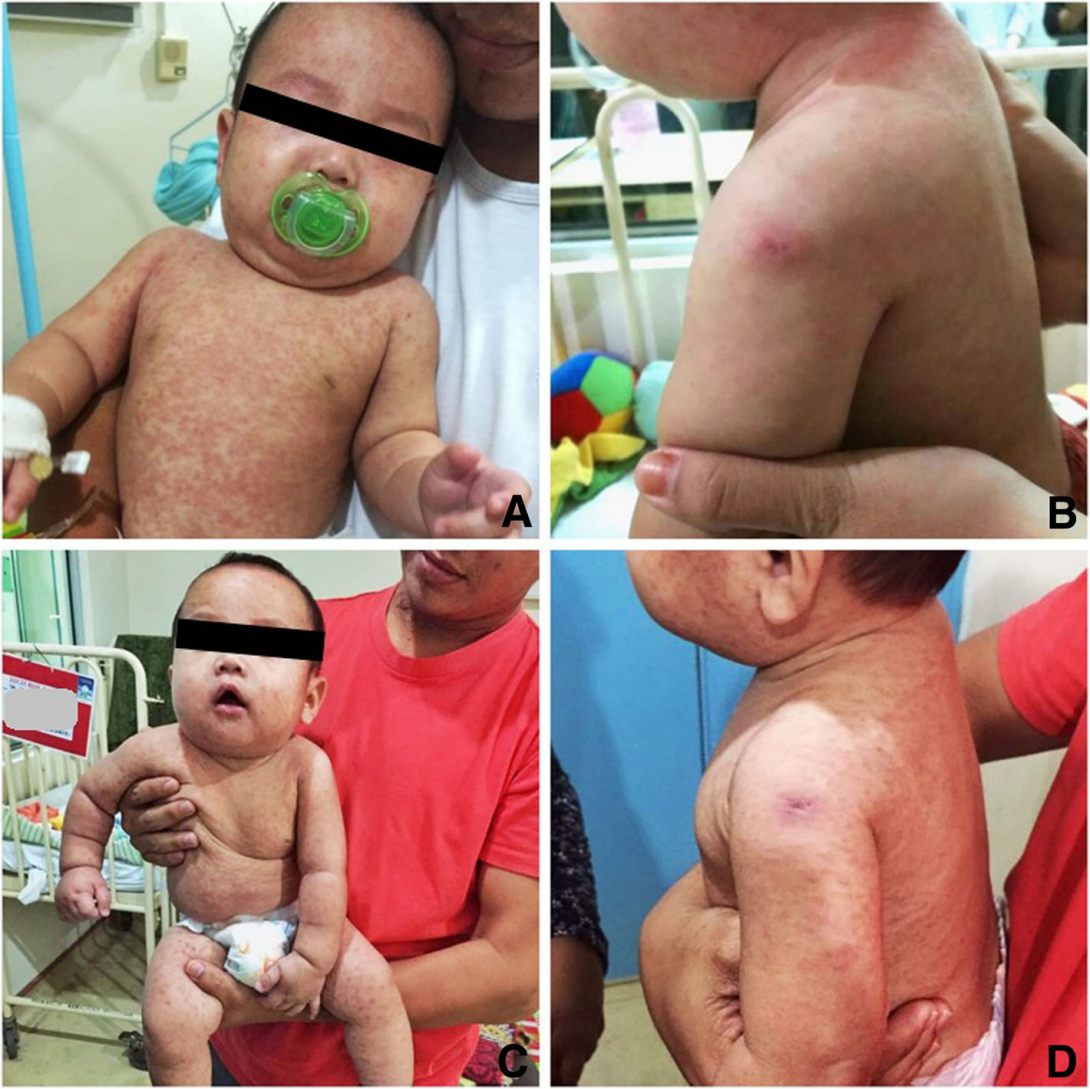
Serial images showing the typical measles rash and the erythema/induration at the BCG scar. Images **a** and **b** (day 5 of illness) show the initial skin lesions while images **c** and **d** (day 7 of illness) show resolution of the lesions with hyperpigmentation of the skin

On admission, he looked unwell, was febrile (temperature 38.5 °C) but had normal vital signs (pulse rate 125/min, respiratory rate 48/min). He had an erythematous maculopapular rash involving mainly the face, neck and trunk, with some areas of confluence (Fig. 1a). In addition, a well-defined erythematous, indurated area measuring 2.5 cm in diameter was seen at the site of the BCG scar (Fig. 1b). No conjunctivitis, cervical node enlargement, mucosal changes or extremity changes (swelling/erythema of the hands or feet) were found.

Laboratory investigations revealed a mild anaemia (haemoglobin 10.9 g/dL) with normal white blood cell (6.5 X 10^9^ cells/L) and platelet counts (252 X 10^9^/L). Both the C-reactive protein (< 6 mg/dL) and the erythrocyte sedimentary rate (10 mm/hour) were normal. Other investigations including liver function tests (albumin 42 g/L; aspartate aminotransferase 41 U/L; alanine aminotransferase 17 U/L) and urine microscopy were also normal. Blood for bacterial culture was negative. A throat swab and urine sample for measles virus isolation, as well as serum for measles antibodies were sent to the regional laboratory.

He was treated with oral vitamin A and intra-venous fluids. His condition began to improve on day 6 of illness, with a resolution of the fever and improvement in feeding and activity. By the 7th day of illness, both the generalized rash as well as the BCG inoculation site erythema and induration appeared to be resolving, leaving some hyperpigmentation (Fig. 1c, d). He was then discharged home.

Follow-up revealed a healthy and thriving boy. No skin desquamation was noted. Echocardiography at 2 and 10 weeks after discharge were normal. A review of the investigation results from the regional laboratory confirmed the diagnosis of measles, with measles virus isolated from the patient’s throat swab and measles-specific antibodies (IgM) present in the serum. Isolation of measles virus from the throat swab was performed using Vero/hSLAM cell lines. Typical cytopathic effect was observed as formation of syncytia, which appeared as large multinucleated cells (giant cells). The cultures were then harvested and the presence of measles virus confirmed by an immunofluorescence assay (Chemicon International, Temecula, USA). Measles-specific antibodies (IgM) in the serum were detected by an indirect enzyme linked immuno-sorbent assay (ELISA) using a commercially available test kit (Siemens Healthcare Diagnostics Products GmbH, Marburg, Germany).

## Discussion

To the best of our knowledge, this is the first case report to describe BCG reactivation in a child with confirmed measles infection. A search of the PubMed/MEDLINE database using the key terms “Bacillus Calmette-Guérin”, “Bacille Calmette-Guérin”, “BCG” and “measles” revealed no reports of similar cases in the published literature. Although the detection of BCG inoculation site erythema and induration did prompt a thorough investigation for KD in our patient, diagnostic criteria for the disease was not met. All clinical and laboratory findings were however consistent with the diagnosis of measles, and a complete and rapid recovery ensued with supportive therapy.

The diagnosis of measles in the patient was confirmed by both measles virus isolation from the throat swab and also detection of measles-specific antibodies (IgM) in the serum. Having been exposed to an unvaccinated relative who had laboratory-confirmed measles, the patient, a 7-month old infant, then developed the classical manifestations and progressed through the typical natural history of measles infection, without developing any other complications. Measles infection is caused by measles virus, a *Morbillivirus* in the family paramyxoviridae [9]. The disease remains an important childhood affliction, causing 89 780 deaths globally in 2016, despite the availability of an effective vaccine [10]. In Malaysia, measles vaccination is given to infants aged 9-months. With a vaccination coverage of 94% in 2016, the incidence of measles in Malaysia was reported to be 5.0 per 100,000 population while the mortality rate from measles was 0.02 per 100,000 population [11]. Although the diagnosis is usually made based on the characteristic clinical manifestations, confirmation of measles infection may be obtained by detection of measles specific IgM antibodies in the serum, and also through measles virus identification from throat-swab and urine specimens [12].

Other than fever, the rash and the BCG inoculation site erythema and induration, no other features of KD were present in the patient. The clinical symptoms and signs resolved without KD specific therapy, with no coronary artery complications detected on follow up. KD is an acute vasculitic syndrome of unknown etiology, occurring mainly in children [13]. As there are no confirmatory tests, the diagnosis of KD is made based on the presence of fever lasting for 5 days or more, and 4 of 5 principal clinical criteria that include conjunctivitis without exudates, cervical lymphadenopathy, polymorphous rash, changes in the lips or oral mucosa, and changes of the extremities [13]. A major challenge in the diagnosis and management of KD, however, is the recognition that some infants present with only 2–3 clinical features, and therefore do not fulfill the diagnostic criteria. Laboratory investigations may be of some use in this situation of incomplete KD [13]. BCG reactivation has repeatedly been described as an important sign in incomplete KD, although it is not included in any of the diagnostic algorithms [14, 15]. For example, in South Korea, 85% of children aged < 1 year who were diagnosed with incomplete KD had erythema, induration or crust formation at the BCG inoculation site [16]. The unexpected finding of BCG reactivation in our patient correctly raised the concern of a possible diagnosis of incomplete KD. Although echocardiography was not performed in the initial phase, no additional supporting clinical or laboratory criteria, other than mild anemia, were found. Reassuringly, the rapid resolution of symptoms precluded any further concerns with regard to the need for KD therapy in the patient.

Apart from those to confirm the measles infection, no laboratory investigations were undertaken to determine if additional viral pathogens were present in the patient and contributed to the development of the BCG reactivation. Human herpes virus 6 infection has been reported in an infant with a viral exanthem presenting with BCG reactivation [17]. In addition, various respiratory viruses including enterovirus, adenovirus, human rhinovirus and coronavirus have been isolated significantly more frequently in children with KD than in controls [18], although no direct association with BCG reactivation have been documented. As viral co-infections have been identified in children with measles [19], a more extensive virology work-up may have been warranted in our patient when the unusual finding of BCG reactivation was discovered.

The mechanism leading to the BCG reactivation in our patient with measles is not known. One possible mechanism is the reactivation and multiplication of live but dormant *Mycobacterium bovis* BCG bacilli that may have been present at the inoculation site, facilitated by immune suppression induced by measles infection. In one previous case report, a severely malnourished 2.5-year old boy developed ulceration of a healed BCG scar and possible disseminated BCG infection 2 weeks after measles infection [20]. Acid-fast bacilli was detected from a swab of the BCG ulcer. This mechanism is, however, unlikely in our patient in view of the rapid onset and resolution of the BCG site erythema/induration with the appearance and resolution of the measles rash. Immune suppression induced by measles would normally be expected to persist for a longer duration [21]. In addition, the lack of malnutrition in the patient, the otherwise uncomplicated measles course, and the absence of secondary infections normally seen in severe immunosuppressed states further renders this mechanism unlikely. Another possible mechanism for the BCG reactivation, which appears more probable, is an immune-mediated reaction. Cross-reactivity between specific epitopes of mycobacterial and human heat shock proteins (HSP), in particular mycobacterial HSP 65 and human homologue HSP 63, have been postulated as the cause of BCG reactivation in children with Kawasaki disease [22, 23]. HSP are ubiquitous molecules present in all organisms, including humans, and mediate important functions in protein folding, assembly and transport essential for cell survival [24]. Synthesis of HSP are increased during conditions of cellular stress, including infection, ischaemia and other physical stresses. In humans, increased production and serum concentrations of HSP, especially HSP 70, have been reported during viral infections, including measles [25, 26]. As homology between HSP 70 and mycobacterial antigens have been demonstrated [27], a similar cross-reactivity as thought to occur in children with Kawasaki disease could possibly also explain the BCG reactivation in our patient. These mechanisms are especially intriguing as measles infections typically cause suppression of delayed type hypersensitivity and anergy to tuberculin [9].

## Conclusions

We report the rare finding of BCG reactivation in a child with confirmed measles infection. Although BCG reactivation is known to be an extremely important and highly specific clinical manifestation of KD, this case suggests that BCG reactivation may also occur in other childhood diseases or infections. A thorough search for an infective aetiological agent may be indicated in children presenting with this clinical finding.

## Data Availability

All relevant data are included within the manuscript.

## Abbreviations

BCG: Bacillus Calmette-Guérin
HSP: Heat shock proteins
IgM: Immunoglobulin M
KD: Kawasaki disease
